# Conserved quantities of Euler-Lagrange system via complex Lagrangian

**DOI:** 10.1016/j.heliyon.2023.e17059

**Published:** 2023-06-09

**Authors:** M. Umar Farooq, Anum Naseem, C. Wafo Soh

**Affiliations:** a Department of Basic Sciences & Humanities, College of E & ME, National University of Sciences and Technology (NUST), H-12, Islamabad, Pakistan; b Department of Mathematics, Faculty of Engineering and Computer Sciences, National University of Modern Languages (NUML), H-9, Islamabad, Pakistan; c Department of Mathematical and Statistical Sciences, Jackson State University, 1400 J R Lynch Street, Jackson, MS, 39257, USA

**Keywords:** Euler-Lagrange system, Noether-like operator, Conserved quantity

## Abstract

In this work we use complex Lagrangian technique to obtain Noether-like operators and the associated conserved quantities of an Euler-Lagrange (EL) system. We show that the three new conserved quantities namely, Noether conserved quantity, Lie conserved quantity and Mei conserved quantity reported by Fang et al. [1] for an EL-system and even more in numbers by Nucci [2] can also be obtained via complex variational formalism. Generally, a linear system of EL-equations possesses maximum 8-dimensional algebra of Noether symmetries and Noether's theorem yields related 8-first integrals. However, our methodology produces 10 Noether-like operators and 10 corresponding invariant quantities for the underlying system of equations. Among those ten first integrals, three (as named above) are reminiscent to those found in [1]. In addition, from the remaining list of conserved quantities several are similar to those reported in [2]. Moreover, the current study presents an alternative approach to compute invariant quantities of EL-systems and leads to interesting and fascinating results.

## 1 Introduction

It is quite known that if an ordinary differential equation (ODE) is invariant under a Lie point symmetry then it can be utilized to integrate or to reduce the order of that equation. In this regard, the possession of one Lie point symmetry enables one to decrease the order of given ODE by one while two Lie point symmetries assist to reduce the order by two. On the other hand if an EL-equation admits a Noether symmetry, then it helps in double reduction of order as well as generating the related conserved quantity via Noether's theorem [3]. In the last couple of years the study of relationship between symmetries and conservation laws has received considerable attention. A number of techniques have been proposed to derive conserved quantities of ODEs of practical interest. They include Noether's method [3], variational method [4], integrating factor method [5], nonlocal conservative method [6], [7], multiplier method [8] and partial Lagrangian method [9]. Some other related work on the construction of conserved quantities for dynamical systems can also be found in [10], [11], [12]. Moreover, the authors [13], [14], [15], [16] have proposed a complex variable approach to investigate the group theoretic properties as well as to determine conservation laws of systems of two EL-equations.

In a recent paper, Fang et al., [1] investigated a new type of conserved quantity which is induced by symmetries of Lagrange system. They found Lie conserved quantity, Noether conserved quantity and Mei conserved quantity for two-dimensional EL-system. Later on, Nucci [2] moved one step forward and applied Noether's theorem to accomplish the results by finding complete set of Noether symmetries and the associated invariant quantities of the system studied in [1].

In this letter, our aim is to construct conserved quantities of an EL-system considered in [1], [2] by applying a well known and legitimate complex Lagrangian approach introduced in [13], [14], [15], [16]. We establish the results reported by Fang et al., [1] and Nucci [2] for the following two-dimensional system of EL-equations

(1) $$\overset{¨}{y_{1}}(t)=-ay_{1}+bt,\;\;\;\;\;\;\overset{¨}{y_{2}}(t)=-ay_{2},\;\;\;where\;\;\;a=\frac{k}{m},\;\;\;b=\frac{1}{m}.$$

As we proceed we see that how our approach can simply and usefully be applied to study group theoretic properties as well as to yield conserved quantities of many EL-systems of type (1).

The structure of the forthcoming discussion is as follows: The next section is devoted to expressions on Noether-like operators and first integrals for system of equations. In section 3, we determine Noether-like operators and associated conserved quantities of (1). We find that these are come out to be the Lie, Noether and Mei conserved quantities as determined in [1], [2]. Finally, we give concluding remarks in section 4.

## 2 Some basics on Noether-like operators and first integrals

We commence with few basic terminologies on Noether-like operators and related expressions to work out first integrals (conservation laws) for two-dimensional EL-systems. We set a standard adaptable criterion to deduce conditions and theorems which will be used as means to determine useful operators and their effectiveness in the production of conserved quantities. For comprehensive details one is referred to read the papers [13], [14], [15], [16] (and the references therein).

Consider the following general system of second-order ODEs

(2) $$\overset{¨}{y_{i}}=f_{i}(t,y_{1},\overset{˙}{y_{1}},y_{2},\overset{˙}{y_{2}}),\;\;\;\;\;\;i=1,2.$$

We assume that the above system (2) is expressible as a single equation, i.e., the transformations, $y(t)=y_{1}(t)+iy_{2}(t)$ and $f(t,y,\overset{˙}{y})=f_{1}(t,y_{1},\overset{˙}{y_{1}},y_{2},\overset{˙}{y_{2}})+if_{2}(t,y_{1},\overset{˙}{y_{1}},y_{2},\overset{˙}{y_{2}})$ exist which connect the above system (2) to a single complex ODE

(3) $$\overset{¨}{y}=f(t,y,\overset{˙}{y}).$$

We also make another assumption that the base Eq. (3) appears in a variational form. So, by utilizing the complex variational structure of Eq. (3), we shall elucidate interesting results for the corresponding system (2). It is evident that above system (2) may or may not necessarily be connected to (3). However, in our case we are lucky enough to transform the system (2) to a single complex scalar Eq. (3). Moreover, we stress here that the salient features of two dimensional system (1) can be studied with the help of a single related equation of the form (3).

To proceed we assume that there exist a complex Lagrangian $L(t,q,\overset{˙}{q})=L_{1}(t,q_{1},\overset{˙}{q_{1}})+iL_{2}((t,q_{1},\overset{˙}{q_{1}}))$ whose insertion in the complex EL-equation provides (3) [14]. Therefore, we find two copies of real Lagrangians $L_{1}$ and $L_{2}$ whose utilization in the following EL-type equations yields the system (2)

(4) $$\begin{array}{cc} \frac{\partial L_{1}}{\partial y_{1}}+\frac{\partial L_{2}}{\partial y_{2}}-\frac{d}{dx}(\frac{\partial L_{1}}{\partial \overset{˙}{y_{1}}}+\frac{\partial L_{2}}{\partial \overset{˙}{y_{2}}}) & =0,\\ \frac{\partial L_{2}}{\partial y_{1}}-\frac{\partial L_{1}}{\partial y_{2}}-\frac{d}{dx}(\frac{\partial L_{2}}{\partial \overset{˙}{y_{1}}}-\frac{\partial L_{1}}{\partial \overset{˙}{y_{2}}}) & =0. \end{array}$$

**Definition.** For the optimal functions $L_{1}$ and $L_{2}$, the operators ${\text{X}}_{1}=\xi_{1}(t,y_{1},y_{2})\frac{\partial }{\partial t}+\eta_{1}(t,y_{1},y_{2})\frac{\partial }{\partial y_{1}}+\eta_{2}(t,y_{1},y_{2})\frac{\partial }{\partial y_{2}}$ and ${\text{X}}_{2}=\xi_{2}(t,y_{1},y_{2})\frac{\partial }{\partial t}+\eta_{2}(t,y_{1},y_{2})\frac{\partial }{\partial y_{1}}-\eta_{1}(t,y_{1},y_{2})\frac{\partial }{\partial y_{2}}$ are named as Noether-like operators of (2) if the subsequent conditions hold

(5) $${\text{X}}_{1}^{\left(1\right)}L_{1}-{\text{X}}_{2}^{\left(1\right)}L_{2}+(D_{t}\xi_{1})L_{1}-(D_{t}\xi_{2})L_{2}=D_{t}A_{1},{\text{X}}_{1}^{\left(1\right)}L_{2}-{\text{X}}_{2}^{\left(1\right)}L_{1}+(D_{t}\xi_{1})L_{2}+(D_{t}\xi_{2})L_{1}=D_{t}A_{2},$$

where $A_{1}$ and $A_{2}$ are gauge functions and $D_{t}=\frac{d}{dt}$.

**Noether-like theorem.** The Noether-like operators ${\text{X}}_{1}$ and ${\text{X}}_{2}$ related to the Lagrangians functions $L_{1}$ and $L_{2}$ for (2), the following expressions yield two invariant quantities

(6) $$C_{1}=\xi_{1}L_{1}-\xi_{2}L_{2}+\frac{\partial L_{1}}{\partial \overset{˙}{y_{1}}}(\eta_{1}-\overset{˙}{y_{1}}\eta_{1}-\overset{˙}{y_{2}}\xi_{2})-\frac{\partial L_{2}}{\partial \overset{˙}{y_{1}}}(\xi_{2}-\overset{˙}{y_{1}}\xi_{2}-\overset{˙}{y_{2}}\xi_{1})-A_{1},C_{2}=\xi_{1}L_{2}+\xi_{2}L_{1}+\frac{\partial L_{2}}{\partial \overset{˙}{y_{1}}}(\eta_{1}-\overset{˙}{y_{1}}\xi_{1}-\overset{˙}{y_{2}}\xi_{2})+\frac{\partial L_{1}}{\partial \overset{˙}{y_{1}}}(\eta_{2}-\overset{˙}{y_{1}}\xi_{2}-\overset{˙}{y_{2}}\xi_{1})-A_{2}.$$

## 3 Lie, Noether and Mei conserved quantities for two-dimensional EL-system

After presenting key expressions of Noether-like operators and Noether-like theorem we deduce known important results on Lie, Noether and Mei conserved quantities of a two-dimensional EL-system (1) in a simple and nontrivial way.

We commence our discussion by observing that the system (1) is transformable to a single complex equation

(7) $$\overset{¨}{y}=-ay+t,\;\;\;\;a=\frac{k}{m},\;\;\;b=\frac{1}{m}$$

under the transformation $y(t)=y_{1}(t)+iy_{2}(t)$. Furthermore, it is readily verified that the Eq. (7) admits the complex Lagrangian, $L=\frac{1}{2}{\overset{˙}{y}}^{2}-\frac{a}{2}y^{2}+bty$, whose splitting into real and imaginary parts yields

(8) $$\begin{array}{cc} L_{1} & =\frac{1}{2}({\overset{˙}{y_{1}}}^{2}-{\overset{˙}{y_{2}}}^{2})-\frac{a}{2}(y_{1}^{2}-y_{2}^{2})+bty_{1},\\ L_{1} & =\overset{˙}{y_{1}}\overset{˙}{y_{2}}-a(y_{1}y_{2})+bty_{2}. \end{array}$$

Interestingly, the insertion of above pair of real Lagrangians in Eqs. (4) and (5) yields respectively the system (1) and the following 10-Noether like operators

(9) $$X_{1}=\frac{\partial }{\partial t}+\frac{b}{a}\frac{\partial }{\partial y_{1}},X_{2}=\frac{b}{a}\frac{\partial }{\partial y_{2}},X_{3}=\cos(\sqrt{a}t)\frac{\partial }{\partial y_{1}},X_{4}=\cos(\sqrt{a}t)\frac{\partial }{\partial y_{2}},X_{5}=\sin(\sqrt{a}t)\frac{\partial }{\partial y_{1}},X_{6}=-\sin(\sqrt{a}t)\frac{\partial }{\partial y_{2}},X_{7}=\cos(2\sqrt{a}t)\frac{\partial }{\partial t}+(\frac{b}{a}\cos(2\sqrt{a}t)+\frac{b}{\sqrt{a}}t\sin(2\sqrt{a}t)-\sqrt{a}y_{1}\sin(2\sqrt{a}t))\frac{\partial }{\partial y_{1}}-\sqrt{a}y_{2}\sin(2\sqrt{a}t)\frac{\partial }{\partial y_{2}},X_{8}=-(\frac{b}{a}\cos(2\sqrt{a}t)+\frac{b}{\sqrt{a}}t\sin(2\sqrt{a}t)-\sqrt{a}y_{1}\sin(2\sqrt{a}t))\frac{\partial }{\partial y_{2}}-\sqrt{a}y_{2}\sin(2\sqrt{a}t)\frac{\partial }{\partial y_{1}},X_{9}=\sin(2\sqrt{a}t)\frac{\partial }{\partial t}+(\frac{b}{a}\sin(2\sqrt{a}t)-\frac{b}{\sqrt{a}}t\cos(2\sqrt{a}t)+\sqrt{a}y_{1}\cos(2\sqrt{a}t))\frac{\partial }{\partial y_{1}}+\sqrt{a}y_{2}\cos(2\sqrt{a}t)\frac{\partial }{\partial y_{2}},X_{10}=\sqrt{a}y_{2}\cos(2\sqrt{a}t)\frac{\partial }{\partial y_{1}}-(\frac{b}{a}\sin(2\sqrt{a}t)-\frac{b}{\sqrt{a}}t\cos(2\sqrt{a}t)+\sqrt{a}y_{1}\cos(2\sqrt{a}t))\frac{\partial }{\partial y_{2}}.$$

We point out here that a two-dimensional system of linear second-order EL-equations admits maximum 8-dimensional algebra of Noether symmetries as found in [2]. However, we notice that the complex Lagrangian formalism provides 10- Noether-like operators amongst which some are the Noether symmetries of (1). For instance, the Noethet-like operator $X_{1}$ coincides with $\Gamma_{10}$ while other operators $X_{3},X_{4},X_{5},X_{6}$ are identical to Noether symmetry generators $\Gamma_{14},\Gamma_{6},\Gamma_{15},\Gamma_{7}$, respectively (given in [2]). Finally, making use of above 10-operators given in (9) along with the Lagrangians (8) in the Noether-like theorem (6), we determine respectively the following ten conserved quantities

(10) $$C_{1}=\frac{b}{a}\overset{˙}{y_{1}}-\frac{1}{2}({\overset{˙}{y_{1}}}^{2}-{\overset{˙}{y_{2}}}^{2})-\frac{a}{2}({y_{1}}^{2}-{y_{2}}^{2})+bty_{1}-\frac{b}{2\sqrt{a}}t^{2},C_{2}=\frac{b}{a}\overset{˙}{y_{2}}-\overset{˙}{y_{1}}\overset{˙}{y_{2}}-ay_{1}y_{2}+bty_{2},C_{3}=(\frac{a}{b}\overset{˙}{y_{1}}-1)\cos(\sqrt{a}t)+\sqrt{a}(\frac{a}{b}y_{1}-t)\sin(\sqrt{a}t),C_{4}=\overset{˙}{y_{2}}\cos(\sqrt{a}t)+\sqrt{a}y_{2}\sin(\sqrt{a}t),C_{5}=(\frac{a}{b}\overset{˙}{y_{1}}-1)\sin(\sqrt{a}t)-\sqrt{a}(\frac{a}{b}y_{1}-t)\cos(\sqrt{a}t),C_{6}=\overset{˙}{y_{2}}\sin(\sqrt{a}t)-\sqrt{a}y_{2}\cos(\sqrt{a}t),C_{7}=\cos(2\sqrt{a}t)[\frac{1}{2}({\overset{˙}{y_{1}}}^{2}-{\overset{˙}{y_{2}}}^{2})-\frac{a}{2}(y_{1}^{2}-y_{2}^{2})+bty_{1}]+[\frac{b}{a}\overset{˙}{y_{1}}\cos(2\sqrt{a}t)-\sqrt{a}\sin(2\sqrt{a}t)\times (y_{1}\overset{˙}{y_{1}}-y_{2}\overset{˙}{y_{2}})+\frac{b}{\sqrt{a}}t\overset{˙}{y_{1}}\sin(2\sqrt{a}t)-({\overset{˙}{y_{1}}}^{2}-{\overset{˙}{y_{2}}}^{2})\cos(2\sqrt{a}t)]+a\cos(2\sqrt{a}t)(y_{1}^{2}-y_{2}^{2})-2bty_{1}\cos(2\sqrt{a}t)+\frac{b}{\sqrt{a}}y_{1}\sin(2\sqrt{a}t)+\frac{b^{2}}{2a}t^{2}\cos(2\sqrt{a}t)-\frac{b^{2}}{2a^{2}}\cos(2\sqrt{a}t)-\frac{b^{2}}{a^{3/2}}\times t\sin(2\sqrt{a}t)+\frac{b^{2}}{2a^{2}},C_{8}=\cos(2\sqrt{a}t)(\overset{˙}{y_{1}}\overset{˙}{y_{2}}-ay_{1}y_{2}+bty_{2})+[\frac{b}{a}\overset{˙}{y_{2}}\cos(2\sqrt{a}t)-\sqrt{a}\sin(2\sqrt{a}t)\times (y_{1}\overset{˙}{y_{2}}+y_{2}\overset{˙}{y_{1}})+\frac{b}{\sqrt{a}}t\overset{˙}{y_{2}}\sin(2\sqrt{a}t)-2\overset{˙}{y_{1}}\overset{˙}{y_{2}}\cos(2\sqrt{a}t)]+2ay_{1}y_{2}\cos(2\sqrt{a}t)-2bty_{2}\cos(2\sqrt{a}t)+\frac{b}{\sqrt{a}}y_{2}\sin(2\sqrt{a}t),C_{9}=\sin(2\sqrt{a}t)[\frac{1}{2}({\overset{˙}{y_{1}}}^{2}-{\overset{˙}{y_{2}}}^{2})-\frac{a}{2}(y_{1}^{2}-y_{2}^{2})+bty_{1}]+[\frac{b}{a}\overset{˙}{y_{1}}\sin(2\sqrt{a}t)+\sqrt{a}\cos(2\sqrt{a}t)\times (y_{1}\overset{˙}{y_{1}}-y_{2}\overset{˙}{y_{2}})-\frac{b}{\sqrt{a}}t\cos(2\sqrt{a}t)\overset{˙}{y_{1}}-({\overset{˙}{y_{1}}}^{2}-{\overset{˙}{y_{2}}}^{2})\sin(2\sqrt{a}t)]-a\sin(2\sqrt{a}t)(y_{1}^{2}-y_{2}^{2})+2bt\sin(2\sqrt{a}t)y_{1}-\frac{b}{\sqrt{a}}\cos(2\sqrt{a}t)y_{1}-\frac{b^{2}}{2a}t^{2}\sin(2\sqrt{a}t)+\frac{b^{2}}{2a^{2}}\sin(2\sqrt{a}t)+\frac{b^{2}}{a^{3/2}}\times t\cos(2\sqrt{a}t)+\frac{b^{2}}{2a^{2}}\sin(2\sqrt{a}t),C_{10}=\sin(2\sqrt{a}t)(\overset{˙}{y_{1}}\overset{˙}{y_{2}}-ay_{1}y_{2}+bty_{2})+[\frac{b}{a}\overset{˙}{y_{2}}\sin(2\sqrt{a}t)+\sqrt{a}\cos(2\sqrt{a}t)\times (y_{1}\overset{˙}{y_{2}}+y_{2}\overset{˙}{y_{1}})-\frac{b}{\sqrt{a}}t\cos(2\sqrt{a}t)\overset{˙}{y_{2}}-2\overset{˙}{y_{1}}\overset{˙}{y_{2}}\sin(2\sqrt{a}t)]-2a\sin(2\sqrt{a}t)y_{1}y_{2}+2bt\sin(2\sqrt{a}t)y_{2}-\frac{b}{\sqrt{a}}\cos(2\sqrt{a}t)y_{2}.$$

So, the complex Lagrangian formalism produces 10-Noether-like operators and corresponding 10-conserved quantities unlike the case of classical Noether's approach which provides only 8-Noether symmetries and 8-conserved quantities for the underlying system of EL-equations. Here all the above quantities $C_{1},C_{2},...,C_{10}$ satisfy the condition to become conserved quantities of the prescribed system (1). Here, an important conclusions can be acclaimed in comparison to the results established in [1], [2]. For instance, the conserved quantity $C_{1}$ corresponds to Lie conserved quantity as determined in [1] (Eq. 37) and $I_{10}$ in Eq. (5) in [2], $C_{2}$ corresponds to Mei conserved quantity in [1] (Eq. (46)) and in [2] (Eq. (9)) and $C_{3}$ is the Noether conserved quantity as determined in [2]. Thus we enter into an interesting and fascinating area of research that many classical results can be achieved with the help of complex Lagrangian technique along with some additional information in the form of useful operators and conserved quantities.

Furthermore, it is generally believed that the availability of maximum numbers of invariant quantities often considered a first step towards finding the solution of underlying system. Remarkably, the elimination of $\overset{˙}{q_{1}}$ from the quantities $C_{3}$ and $C_{5}$ given in Eq. (10) provides

(11) $$y_{1}(t)=\frac{b}{a}[\frac{1}{\sqrt{a}}(C_{3}\sin(2\sqrt{a}t)+C_{5}\cos(2\sqrt{a}t))+t].$$

Similarly, by removing $\overset{˙}{q_{2}}$ term from $C_{4}$ and $C_{6}$, one obtains the following solution

(12) $$y_{2}(t)=\frac{1}{\sqrt{a}}[C_{4}\sin(2\sqrt{a}t)+C_{6}\cos(2\sqrt{a}t)].$$

Importantly, it can be checked that the above functions $y_{1}$ and $y_{2}$ determined in Eqs. (11) and (12) are the general solutions of the master system, namely Eq. (1).

## 4 Conclusion

We have addressed the problem of finding the Lie conserved quantity, Noether conserved quantity and Mei conserved quantity of a system of two linear second-order EL-equations by complex Lagrangian approach. The technique elegantly exploits Noether-like operators in order to establish the results as communicated in literature. In general, a linear system of EL-equations possesses maximum 8-dimensional algebra of Noether symmetries and have 8-first integrals. But here, we have seen that our technique yields 10 Noether-like operators and 10 first integrals. Among those 10 conserved quantities, some are the Lie conserved quantity, Mei conserved quantity and Noether conserved quantity which are reminiscent to those reported in [1], [2]. We have also shown that the availability of maximum number of conserved quantities becomes advantageous to reach at the general solution of the underlying system of EL-equations. We conclude with remark that the complex Lagrangian formalism can be efficiently applied to divulge many known results and to probe many additional characteristics of practical problems depicted in the form of systems of EL-equations. An other important aspect of first integrals $C_{1},C_{2},...,C_{10}$ could be thought as which of these satisfy condition of other conserved quantities. For instance, which of them are the Hojman conserved quantities is a subject of future research.

## CRediT authorship contribution statement

M. Umar Farooq: Conceived and designed the experiments; Performed the experiments; Analyzed and interpreted the data; Contributed reagents, materials, analysis tools or data; Wrote the paper.

Anum Naseem: Conceived and designed the experiments; Analyzed and interpreted the data; Contributed reagents, materials, analysis tools or data.

C. Wafo Soh: Contributed reagents, materials, analysis tools or data.

## Declaration of Competing Interest

It is stated that all authors of the paper titled “Conserved quantities of Euler-Lagrange system via complex Lagrangian” have no conflict of interests whatsoever.

## Data Availability

No data was used for the research described in the article.
